# Does order matter? Investigating the effect of sequence on glance duration during on-road driving

**DOI:** 10.1371/journal.pone.0171730

**Published:** 2017-02-03

**Authors:** Joonbum Lee, Shannon C. Roberts, Bryan Reimer, Bruce Mehler

**Affiliations:** 1 MIT AgeLab and New England University Transportation Center, Cambridge, Massachusetts, United States of America; 2 Department of Mechanical and Industrial Engineering, University of Massachusetts Amherst, Amherst, Massachusetts, United States of America; Beihang University, CHINA

## Abstract

Previous literature has shown that vehicle crash risks increases as drivers’ off-road glance duration increases. Many factors influence drivers’ glance duration such as individual differences, driving environment, or task characteristics. Theories and past studies suggest that glance duration increases as the task progresses, but the exact relationship between glance sequence and glance durations is not fully understood. The purpose of this study was to examine the effect of glance sequence on glance duration among drivers completing a visual-manual radio tuning task and an auditory-vocal based multi-modal navigation entry task. Eighty participants drove a vehicle on urban highways while completing radio tuning and navigation entry tasks. Forty participants drove under an experimental protocol that required three button presses followed by rotation of a tuning knob to complete the radio tuning task while the other forty participants completed the task with one less button press. Multiple statistical analyses were conducted to measure the effect of glance sequence on glance duration. Results showed that across both tasks and a variety of statistical tests, glance sequence had inconsistent effects on glance duration—the effects varied according to the number of glances, task type, and data set that was being evaluated. Results suggest that other aspects of the task as well as interface design effect glance duration and should be considered in the context of examining driver attention or lack thereof. All in all, interface design and task characteristics have a more influential impact on glance duration than glance sequence, suggesting that classical design considerations impacting driver attention, such as the size and location of buttons, remain fundamental in designing in-vehicle interfaces.

## Introduction

Driving is an inherently multi-faceted activity that involves key sensing, cognitive, and manipulative resources. Keeping a driver’s hands, mind, eyes, etc. involved in operational activities and threat detection are central to safety in today’s largely manually controlled automobiles. While each sensory system has varying importance at different points of a journey, if drivers take their eyes off of the road for substantial periods of time, risk of a safety critical events has been shown to increase [1–3]. As such, it is paramount to understand how glance behavior may change over the course of a drive with concurrent engagement in non-driving related secondary tasks that typify the modern driving experience.

Given recent advances in vehicle technology, secondary tasks that distract drivers have garnered much attention. Oftentimes, interacting with a Driver Vehicle Interface (DVI) requires multiple and frequent long off-road glances. As such, understanding how the type and structure of different secondary tasks affect glance behavior to inform the effective management of this scarce attentional resource is critical to minimizing the risk of safety critical events. Broadly speaking, secondary multi-modal tasks can involve varying degrees of input (vocal, manipulative, etc.), output (visual, auditory, etc.), and processing resources. Multiple resource theory [4,5] suggest “costs” where resource demands overlap. For instance, secondary tasks that have an auditory component reduce the driver’s bandwidth with respect to hearing the road (e.g., listening to loud music while driving can impede the drivers’ ability to hear if they are running over rumble strips). Secondary tasks with a cognitive component may impact the degree to which a driver is fully engaged in the processing of threats and making strategic operational decisions (e.g., being lost in thought while driving causing a driver to miss a turn). Manual secondary tasks often take drivers’ hands off of the steering wheel or feet off of the pedals (e.g., reaching for a cell phone can lead drivers to inadvertently steer into another lane). Lastly, visual secondary tasks take drivers’ eyes off of the road (e.g., attending to a child in the back seat that leads to the running of a stop sign).

Each conceptually unique set of glance regions plays a role in the acquisition or depletion of situational understanding. As such, in addition to examining the relationship between task type and glance behavior, it is important to understand if and how drivers thread glances between the road, driving related off-road locations (e.g., mirrors), and non-driving related secondary tasks. Some psychological theories suggest that glance duration might increase as task time increases [6]. In addition, research concerning the use of glance duration to predict driver distraction has also taken advantage of the relationship between previous and current glances via glance history analysis. More specifically, prolonged or an increasing number of glances away from the road have been used as a predictor of driver distraction [3,7,8]. At the same time, glance history analysis has generally not considered the detailed relationship between two consecutive glances. Recent work [9], begins to explore the visualization of these transitions and probabilistic time independent modeling of the relationships through a hidden Markov model. This method, however, does not consider the duration and temporal spacing of glances across a task.

Change in glance behavior over the course of a task has been studied in past research. One study suggested an interaction between playlist length and interface type on drivers’ glances to an MP3 player while scrolling through long playlist, such that there were longer glances away from the road toward the end of long tasks [10]. Another study found that the first two glances while completing in-vehicle tasks were significantly shorter than subsequent glances [11], supporting the interplay between glance sequence and glance duration. However, these early explorations of the phenomena were encumbered by a number of important limitations related to the variability of data and the need for statistical power in small samples. For instance, in [11] different task types were collapsed together (possibly to reduce sample variability) into a single analysis, thereby confounding the effect of task type on glance behavior. In addition, tasks were time limited, that is, participants had to complete tasks within a certain time frame. As such, it is quite plausible to expect that participants’ glance behavior during tasks was influenced by the approach of a perceived or actual time limit.

Outside of these papers [10,11], the effect of glance sequence on glance duration is not well understood. There are several factors that make testing, analysis, and interpretation of sequential effects difficult. For example, the reliability and validity of many eye-tracking systems is uncertain and/or dependent upon experimental setup, thereby casting doubt as to the quality of eye glance data [12,13].

The present research aims to address this research gap by considering several complementary statistical frameworks to investigate the sequential effects of glance behavior while considering variations in task type and task characteristics that may affect off-road glance duration. This study applied multiple statistical methods (i.e., mixed effects modeling and Kolmogorov-Smirnov tests) to allow for different interpretations as to the interplay of glance sequence and glance duration. The source data were collected from 80 participants who completed two types of in-vehicle tasks (radio tuning and navigation address entry) in the same vehicle. The in-vehicle tasks were conducted on a production DVI during actual highway driving.

## Method

This study is a secondary analysis of a subset of data from two larger projects [13,14]. While complete methodological details on the studies can be found in the initial reports, key details related to the analysis presented in this report are summarized here.

The first data collection (henceforth known as data set A) was conducted to assess drivers’ glance behavior, driving performance, and perceived workload while completing a number of tasks with a production-level voice-command system. The second data collection (henceforth known as data set B) aimed to replicate key findings in data set A, but there was an added focus of determining whether two different approaches to training drivers on the DVI—self-guided vs. structured training condition—impacted the overall pattern of interaction; the result showed no statistically significant effects of the training condition. Procedural and technical details of the two studies [13,14] were consistent except where explicitly described in subsequent sections.

### Participants

Recruitment methods and study procedures were approved by Massachusetts Institute of Technology’s institutional review board (IRB) (application number: 804002714) and compensation for participation was provided. Recruitment drew from the greater Boston, MA area using online and newspaper advertisements. Participants were required to meet the following criteria: (a) holding a valid driver’s license for more than three years, (b) driving on average three or more times per week, (c) being in self-reported reasonably good health for their age and meeting a set of health exclusion criteria, (d) clearly understanding and speaking English, (e) having no police reported accident in the past year, (f) not actively using any medications causing drowsiness, and (g) not having been a participant in an MIT AgeLab on-road driving study in the past six months. The two on-road experiments contained a total 124 participants (*n* = 60 for data set A and *n* = 64 for data set B). While these two experiments recruited participants between 20 to 69 years old, data set A (recruitment from March to October, 2012) had two age groups (20–29 and 60–69 years old) and data set B (recruitment from April to October, 2013) had four age groups (18–24, 25–39, 40–54, and 55 and older) to conform to National Highway Traffic Safety Administration (NHTSA)’s recommendations [15]. Therefore, from the pool of 124 participants, 80 subjects were randomly sampled for this analysis to constitute two age groups (20–29 and 60–69 years). Random sampling was used to obtain a sample of 40 participants from each on-road experiment that were equally distributed by age group and gender.

### Apparatus

**Fig 1:**
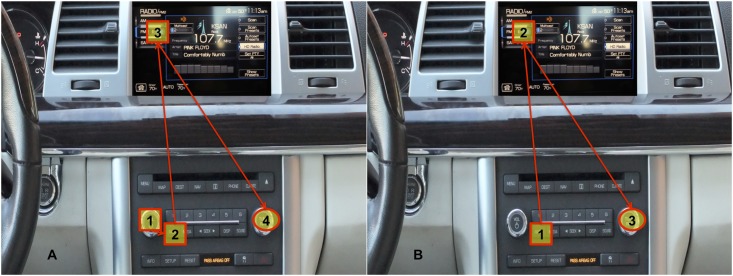
Order in which participants had to press the buttons for completion of the radio tuning task for: (A) data set A; (B) data set B.

A 2010 Lincoln MKS was instrumented and employed to collect on-road data. The DVI included traditional radio controls and a center stack touch screen display (Fig 1). The vehicle was equipped with a customized data acquisition system for time-synchronized recording of vehicle information from the controller area network (CAN) bus and cameras that captured driver behavior and vehicle surroundings. A video recorded the driver’s face at 15fps (640x480) and was used to code drivers’ glance behavior to regions of interest. There were no differences in technical details of the apparatus between data set A and data set B.

### Tasks

For the experiments, drivers were asked to complete a number of in-vehicle DVI tasks (e.g., song selection, navigation entry, radio tuning, and an n-back cognitive demand reference task). The present paper focuses on analysis of data from drivers’ engagement with the visual-manual radio tuning task and auditory-vocal navigation entry task as a representation of two different task types where different glance allocation patterns could be observed.

The basic protocol for the radio tuning task was modeled on the “hard” tuning task employed in the Crash Avoidance Metrics Partnership (CAMP) Driver Workload Metrics project [16], involving several button presses followed by manipulation of a rotary knob to tune to a specified frequency. Comprehensive training was provided while stationary in a parking lot setting prior to on-road assessment that involved two replications of the tuning task (different stations). In data set A, radio tuning required four manual steps: (1) press the power/volume button to turn the system on, (2) press the radio button, (3) select a band button on a touch-sensitive display (e.g., FM1), and (4) rotate the tuning knob to locate a specified station. This sequence of steps is highlighted in picture A of Fig 1. In data set B, the first step, press the power button, was omitted to conform to guidelines subsequently issued by the NHTSA [15] that specify a radio tuning reference task that begins with the radio on. In this form, reaching the task goal only required three manual steps (picture B in Fig 1).

For the navigation entry tasks, there was no protocol difference between the two data sets. The goal of the navigation entry tasks was to enter an alpha-numeric destination addresses using a voice based command system. The task required participants to press a button on the steering wheel to start the voice recognition system and to verbally enter the designated addresses in a series of steps. There were two trials and a different address for each trial (e.g., “77 Massachusetts Avenue, Cambridge, Massachusetts”). The navigation entry task consisted of six steps: (1) press the voice button, (2) say “Destination street address,” (3) say “*City name*,” (4) say “*Street name*,” (5) say “*House number*,” and (6) say “Yes” to confirm the entry. This task is referred to in this paper as an auditory-vocal based multi-modal task since visual-manual components are present even though the primary interaction is through the auditory-vocal channels.

### Procedure

Participants read and signed an IRB approved informed consent form and presented their driver’s license. They then attested to having had their license for more than three years, to driving an average three or more times per week, and to be in self-reported good health. Afterwards, participants completed a pre-experimental questionnaire. Participants were then escorted to the instrumented vehicle and provided detailed training on the DVI tasks to be completed during the first half of the drive. The driving portion of the study was divided into four segments. The first segment consisted of a period of approximately 10 minutes of urban driving to reach interstate highway I-93 and continued north on I-93 for an additional 20 minutes to the I-495 intersection. This created a total adaptation period of approximately 30 minutes of driving prior to the assessment portion of the study. The second segment consisted of driving south on I-495 to exit 19 (rest point) and averaged approximately 40 minutes. The third was from the rest area back north on I-495 to I-93 (approximately 40 minutes) and the fourth was the return on I-93 south (approximately 30 minutes). All DVI tasks were presented in a counter-balanced order during segments two and three. Both tasks were presented sequentially while driving on either I-495 south or I-495 north. The route was consistent across the two experiments.

During the drive, an experimenter, seated in the rear of the vehicle, was responsible for: (a) providing driving directions, (b) ensuring safe vehicle operation, (c) verifying that participants understood and followed instructions, (d) ensuring that recording telemetry was working properly, and (e) making sure that the experiment proceeded according to a predefined script. The data acquisition system supported playing prerecorded audio instructions and the experimenter used a set of F-key presses at predefined points to trigger steps in the experiment. This ensured that primary instructions and tasks were presented to all participants in a consistent manner.

### Glance coding

Two independent research associates manually coded glances from an in-vehicle video of the drivers’ face. Coding was completed using the MIT AgeLab Video Annotator (https://bitbucket.org/agelab/annotator) following a taxonomy [13,14] that included an area of interest around the center stack of the vehicle. Following procedures established in [17], a third independent research associate mediated discrepant glances if: (a) the coders started or ended their coding at different times, (b) a divergent number of glances was coded, (c) the coders identified a divergent target for a glance, or (d) the timing of a coded glance differed by more than 200ms.

### Data analysis

Data reduction and analyses were conducted using the R statistical language [18]. As mentioned, there were two trials for each task, and glance measurements for the trials were calculated for each trial and then averaged together. To test the effect of glance sequence on glance duration, a linear mixed-effect model with glance sequence as a fixed within-subject effect and subject as a random effect was applied. In addition, the Kenward-Roger correction was applied to adjust the *F* statistics and degrees of freedom. As an effect size, both marginal *R*^2^, which describes the proportion of variance explained by the fixed factor alone, and conditional *R*^2^, which describes the proportion explained by the fixed and random factors, are reported in the results section.

## Results

Results are presented below separated by task type. Scenario 1 describes the radio tuning task and Scenario 2 describes the navigation entry task. Within each scenario, results are presented corresponding to the applied statistical methods. First, overall descriptive statistics are provided (e.g., mean duration, glance frequency, etc.). Second, mixed effects modeling and Kolmogorov-Smirnov tests are applied to compare the mean and distributions of the: (a) first set of glances, (b) mean number of glances, and (c) last set of glances. Finally, a comparison of the two Scenarios is presented.

### Scenario 1: Radio tuning tasks

#### Overview of glance measures from radio tuning tasks

Prior to the main analysis, several glance measurements were analyzed and compared across data sets. Average glance duration to the center stack to complete the radio tuning tasks was 1.02 seconds (*M* = 1.02 for data set A, and *M* = 1.02 for data set B). Mean of the shortest glance was 0.46 seconds (*M* = 0.45 for data set A, and *M* = 0.47 for data set B), and mean of the longest glance was 1.89 seconds (*M* = 1.99 for data set A, and *M* = 1.79 for data set B). There was no significant difference for mean single glance duration, *t* (78) = -0.14, *p* = .9, *d* = -0.03, minimum single glance duration, *t* (78) = -0.42, *p* = .8, *d* = 0.09, and maximum single glance duration, *t* (78) = 1.34, *p* = .18, *d* = -0.3, between the two data sets. Percentage of long-duration glances (e.g., over 2 seconds) was 5.01% (4.84% for data set A, and 5.18% for data set B), and there was no significant difference between the two data sets, *t* (78) = -0.2, *p* = .84, *d* = -0.04. Mean number of glances to complete both tasks was 13.36 (*SD* = 4.6; *M* = 12.86 for data set A, and *M* = 13.85 for data set B). There was no significant difference between the data sets in mean number of glances, *t* (78) = -0.96, *p* = .34, *d* = -0.21.

**Fig 2:**
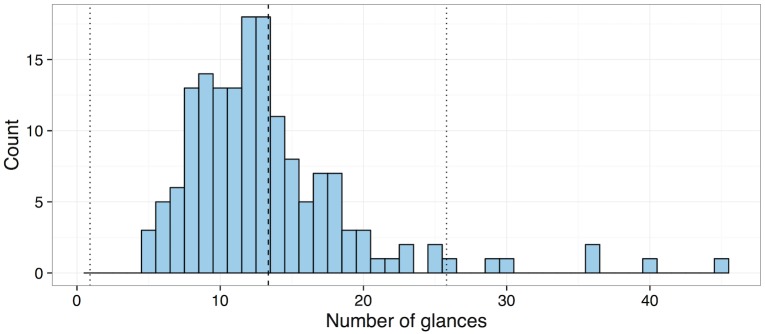
Glance frequency distribution for the radio tuning tasks; the dashed line indicates the mean number of glances and two dotted lines represent two standard deviations in either direction.

Fig 2 shows the distribution of number of glances to complete the radio tuning tasks. Glances that fell outside of the 2 standard deviation range were defined as outliers and excluded for subsequent analyses. Note that seven out of 160 glances were defined as outliers and subsequently excluded. The seven outliers were found only in data set B.

#### Mixed effects modeling

To ensure equality in the analysis across the two data sets, the first six glances were sampled and compared. The first six glances were chosen to analyze given that the minimum number of glances for data set A was five, while for data set B, the minimum was six. To extend the scope of the analysis, the same process was also applied for the first 14 glances (the mean number of glances), and the last six glances.

**Fig 3:**
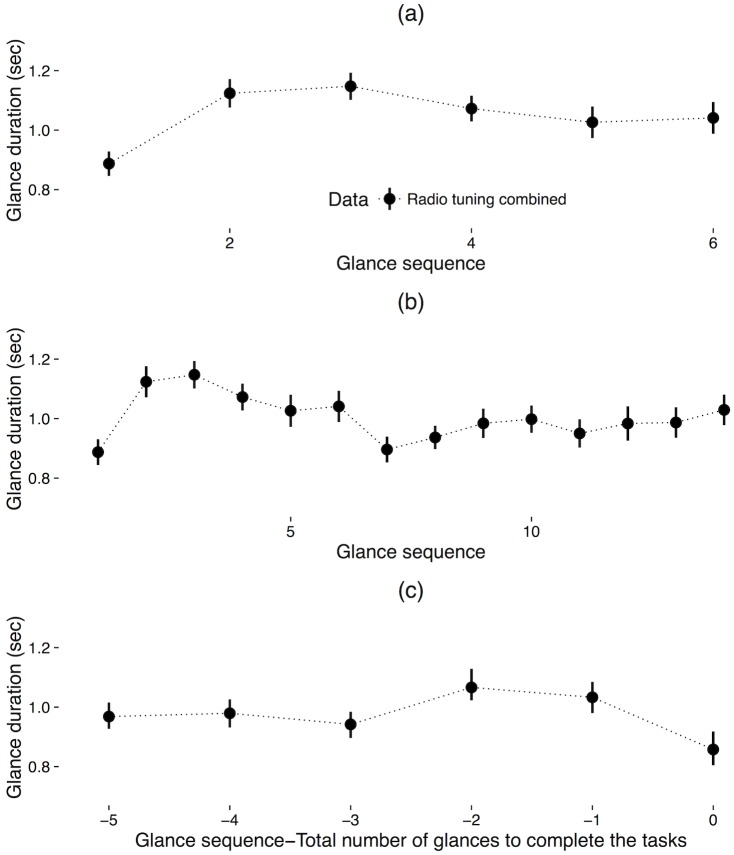
Glance duration as a function of glance sequence for the (a) first six glances, (b) first 14 glances, and (c) last six glances for the radio tuning tasks; error bars represent 1 standard error of the mean.

Plots of mean glance duration across participants for each glance in sequence during the radio tuning tasks are shown in Fig 3. For the first six glances, there was no significant effect of glance sequence on glance duration, *F* (1, 77.89) = 1.1, *p* = .29, marginal *R*^*2*^ = .003, conditional *R*^*2*^ = .27. However, across the first 14 glances, there was a statistically significant effect of glance sequence on glance duration, *F* (1, 72.18) = 5.75, *p* < .05, marginal *R*^*2*^ = .008, conditional *R*^*2*^ = .25, indicating that a one unit increase in glance sequence is associated a 0.01 second decrease in glance duration. For the last six glances, there was no significant effect of glance sequence on glance duration, *F* (1, 77.7) = 0.48, *p* = .49, marginal *R*^*2*^ = .001, conditional *R*^*2*^ = .22.

#### Kolmogorov-smirnov tests

**Fig 4:**
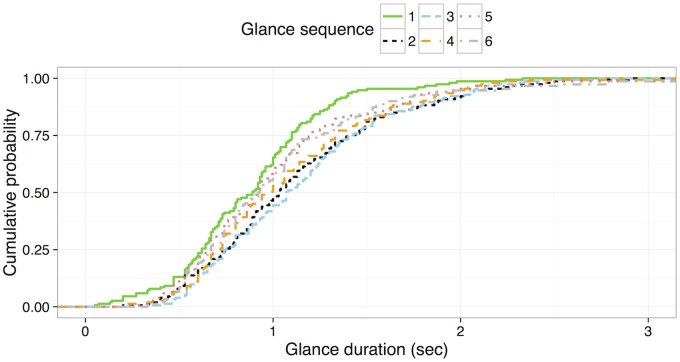
Cumulative probability distribution of glance duration for the first six glances of the radio tuning tasks.

In addition to the mixed effect modeling, Kolmogorov-Smirnov (KS) tests were applied to compare glance sequence to glance duration. While the mixed effect modeling focuses on central tendencies (i.e., the mean), the KS test allows for a comparison of distributions. Fig 4 shows the cumulative probability of glance duration for each of the first six glances.

Table 1 shows *D* statistics (i.e., maximum distance between two cumulative probability curves) of the KS test and a *p* value for each pair of glances. Pairs that rejected the null hypotheses, indicating that two groups were sampled from populations with different distributions, are bolded in the table. Results show that the duration of the first glance is significantly different (i.e., shorter) than the other glances, except the fifth and sixth glance. In addition, the duration of the third glance is significantly different than the fifth and sixth glances. Other than these pairs, there were no significant differences.

**Table 1:** Results of the KS tests examining the first six glances of the radio tuning tasks; each cell entry shows *D* statistics of the KS test for the two glances indicated by the row and column indices; bolded values represent significant differences.

*Glance sequence*					
	**2**	**3**	**4**	**5**	**6**
**1**	***D* = .21** **	***D* = .26** ***	***D* = .18** *	*D* = .11,	*D* = .12,
**2**		*D* = .08	*D* = .08	*D* = .13	*D* = .13
**3**			*D* = .1	***D* = .18** *	***D* = .2** **
**4**				*D* = .1	*D* = .11
**5**					*D* = .05

* *p* < .05,

** *p* < .01,

*** *p* < .001

**Fig 5:**
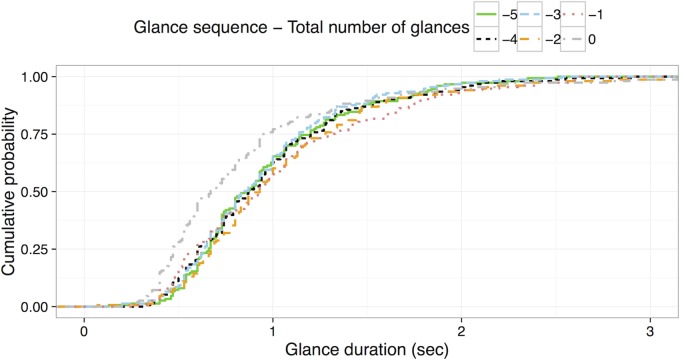
Cumulative probability distribution of glance duration for the last six glances from the radio tuning tasks.

The same analysis was applied for the last six glances (see Fig 5 and Table 2). The results showed that the duration of the last glance is significantly different (i.e., shorter) than the other glances.

**Table 2:** Results of the KS tests for the last six glances of the radio tuning tasks; each cell entry shows *D* statistics of the KS test for the two glances indicated by the row and column indices; bolded values represent significant differences.

*Glance sequence—Total number of glances*					
	**-1**	**-2**	**-3**	**-4**	**-5**
**0**	***D* = .24** ***	***D* = .29** ***	***D* = .24** ***	***D* = .22** **	***D* = .25** ***
**-1**		*D* = .11	*D* = .1	*D* = .09	*D* = .11
**-2**			*D* = .08	*D* = .08	*D* = .1
**-3**				*D* = .05	*D* = .11
**-4**					*D* = .05

* *p* < .05,

** *p* < .01,

*** *p* < .001

**Fig 6:**
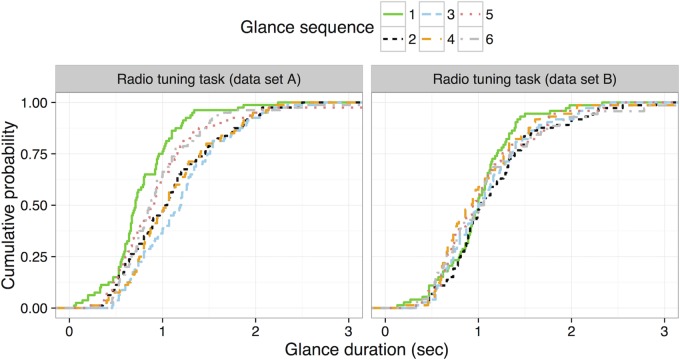
Cumulative probability distribution of glance duration for the first six glances from data set A (on the left) and data set B (on the right) for the radio tuning tasks.

There was one substantive difference in the radio tuning task between two samples; in data set A, participants pushed a large knob at the beginning of the task to turn on the radio while in data set B the task started with the radio already on. To examine whether there was a protocol difference, the two data sets were separately tested with the same technique. Interestingly, data set A showed significantly shorter first glances, whereas data set B showed no differences across glance sequence (see Fig 6, Tables 3 and 4). Specifically, the first glance of data set A was significantly shorter than other glances. In addition, the duration of the fifth and sixth glance was significantly different than the third glance. Data set B showed no significant differences.

**Table 3:** Results of the KS test for the first six glances of data set A for the radio tuning tasks; each cell entry shows *D* statistics of the KS test for the two glances indicated by the row and column indices; bolded values represent significant differences.

*Glance sequence*					
	**2**	**3**	**4**	**5**	**6**
**1**	***D* = .31** ***	***D* = .43** ***	***D* = .35** ***	***D* = .23** *	***D* = .25** *
**2**		*D* = .16	*D* = .13	*D* = .18	*D* = .19
**3**			*D* = .14	***D* = .29** **	***D* = .3** **
**4**				*D* = .2	*D* = .2
**5**					*D* = .08

* *p* < .05,

** *p* < .01,

*** *p* < .001

**Table 4:** Results of the KS test for the first six glances of data set B for the radio tuning tasks; each cell entry shows *D* statistics of the KS test for the two glances indicated by the row and column indices.

*Glance sequence*					
	**2**	**3**	**4**	**5**	**6**
**1**	*D* = .16	*D* = .12	*D* = .18	*D* = .15	*D* = .16
**2**		*D* = .11	*D* = .21	*D* = .18	*D* = .15
**3**			*D* = .12	*D* = .08	*D* = .09
**4**				*D* = .1	*D* = .11
**5**					*D* = .09

* *p* < .05,

** *p* < .01,

*** *p* < .001

### Scenario 2: Navigation entry tasks

Scenario 1 found various results with respect to the effect of glance sequence on glance duration. To test if the patterns were caused by inherit effects of glance sequence or task characteristics (e.g., task type, task structure, etc.), Scenario 2 applied the same set of analyses to a different type of in-vehicle task: auditory-vocal navigation entry.

#### Overview of glance measures from navigation entry tasks

For navigation entry tasks, average glance duration to the center stack was 0.91 seconds (*M* = 0.91 for data set A, and *M* = 0.91 for data set B). Mean of the shortest glance was 0.38 seconds (*M* = 0.39 for data set A, and *M* = 0.36 for data set B) and mean of the longest glance was 1.71 seconds (*M* = 1.75 for data set A, and *M* = 1.68 for data set B). There was no significant difference in mean single glance duration, *t* (78) = -0.05, *p* = .96, *d* = -0.01, minimum single glance duration, *t* (78) = 1.18, *p* = .24, *d* = 0.26, and maximum single glance duration, *t* (78) = 0.41, *p* = .69, *d* = 0.1, between the two data sets. Percentage of long-duration glances (e.g., over 2 seconds) was 1.79% (1.88% for data set A, and 1.71% for data set B); there was no significant difference between the two data sets, *t* (78) = 0.22, *p* = .83, *d* = 0.05. Mean number of glances to the center stack to complete the navigation entry task was 23.1 (*SD* = 18.29; *M* = 22.55 for data set A, and *M* = 23.51 for data set B). There was no significant difference between the data sets in mean number of glances, *t* (78) = -0.27, *p* = .79, *d* = -0.06.

**Fig 7:**
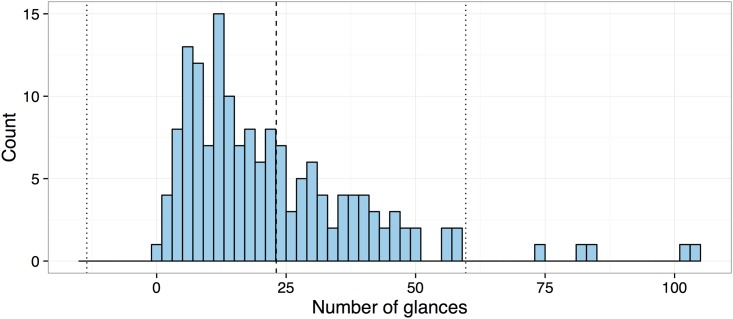
Glance frequency distribution for the navigation entry tasks across both data sets; the dashed line shows mean number of glances and the dotted line represent 2 standard deviations.

Fig 7 shows a distribution of number of glances to complete the navigation entry tasks. Following the same criterion applied in the radio tuning tasks, cases that fell outside of a 2 standard deviation range were defined as outliers and excluded for subsequent analyses. Five of the 160 cases were defined as outliers and excluded: three cases from data set A and two cases from data set B.

#### Mixed effects modeling

**Fig 8:**
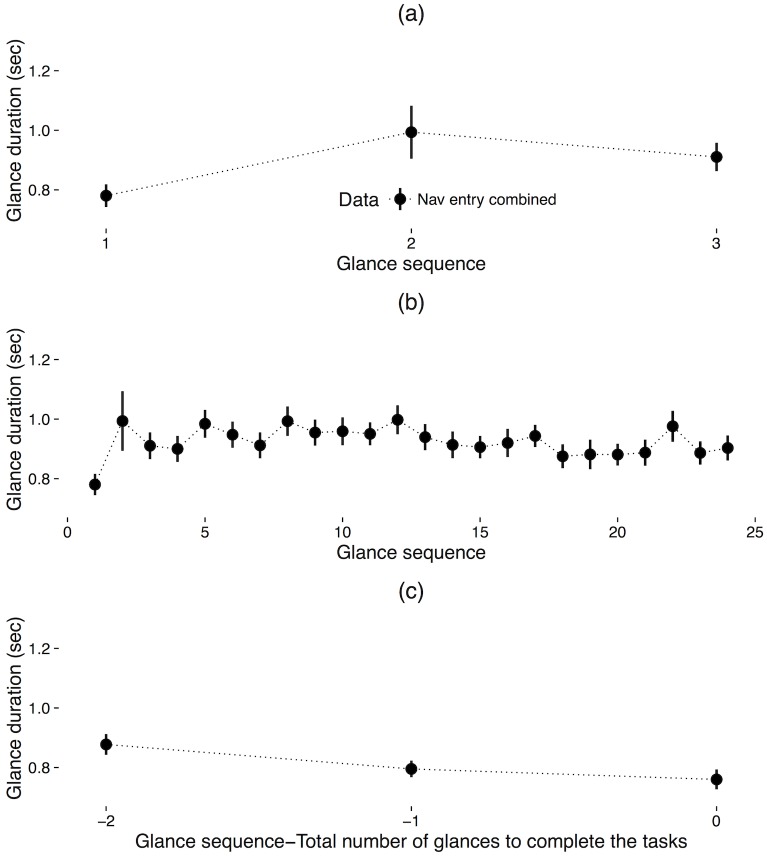
Glance duration for the (a) first three glances, (b) first 24 glances, and (c) last three glances from the navigation entry tasks; error bars represent 1 standard error of the mean.

For the first analysis, the first three glances were compared. The first three glances were chosen to analyze given that the minimum number of glances for data set A was one, while for data set B, the minimum was three. Note that these observations appear as some individuals have been found to complete voice-based multi-modal interfaces with minimal off-road glances while other individuals commit a greater degree of visual attention. Results showed that there was no significant effect of glance sequence on glance duration, *F* (1, 77.62) = 3.55, *p* = .06, marginal *R*^*2*^ = .02, conditional *R*^*2*^ = .11. Second, to extend the scope of the analysis, the same process was also applied for the first 24 glances (the mean number of glances), and the last three glances. For the first 24 glances, results showed that there was no significant effect of glance sequence on glance duration, *F* (1, 56.47) = .13, *p* = .72, marginal *R*^*2*^ = .00005, conditional *R*^*2*^ = .13. For the last three glances, results showed that there was a significant effect of glance sequence on glance duration, *F* (1, 78.16) = 7.78, *p* < .01, marginal *R*^*2*^ = .02, conditional *R*^*2*^ = .21, indicating that a one unit increase in the glance sequence is associated a 0.06 second decrease in the glance duration (Fig 8).

#### Kolmogorov-smirnov tests

**Fig 9:**
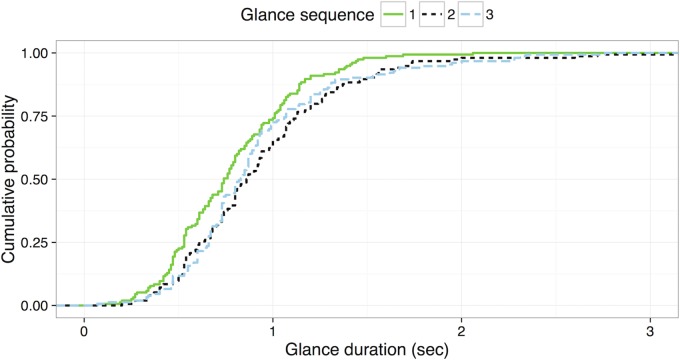
Cumulative probability distribution of glance duration for the first three glances of the navigation entry tasks.

**Fig 10:**
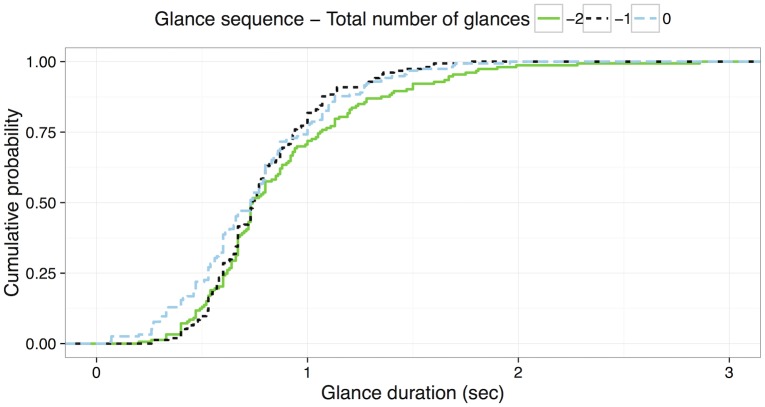
Cumulative probability distribution of glance duration for the last three glances of the navigation entry tasks.

Following the techniques applied in Scenario 1, the KS test was applied to compare the distributions of glance duration from each glance sequence. The distribution for each glance sequence is shown in Fig 9. Table 5 shows *D* statistics of the KS test and *p* values for each pair of glances. Similar to the result from the Scenario 1, the duration of the first glance is significantly different from the other glances for the navigation entry tasks. However, for the last three glances, there was no statistically significant difference between glance sequences (see Fig 10 and Table 6).

**Table 5:** Results of the KS test for the first three glances of the navigation entry tasks; each cell entry shows *D* statistics of the KS test for the two glances indicated by the row and column indices; bolded values represent significant differences.

*Glance sequence*		
	**2**	**3**
**1**	***D* = .17** *	***D* = .17** *
**2**		*D* = .1

* *p* < .05,

** *p* < .01,

*** *p* < .001

**Table 6:** Results of the KS test for the last three glances of the navigation entry tasks; each cell entry shows *D* statistics of the KS test for the two glances indicated by the row and column indices; bolded values represent significant differences.

*Glance sequence—Total number of glances*		
	**-1**	**-2**
**0**	*D* = .14	*D* = .15
**-1**		*D* = .12

* *p* < .05,

** *p* < .01,

*** *p* < .001

### Comparison between two tasks

**Fig 11:**
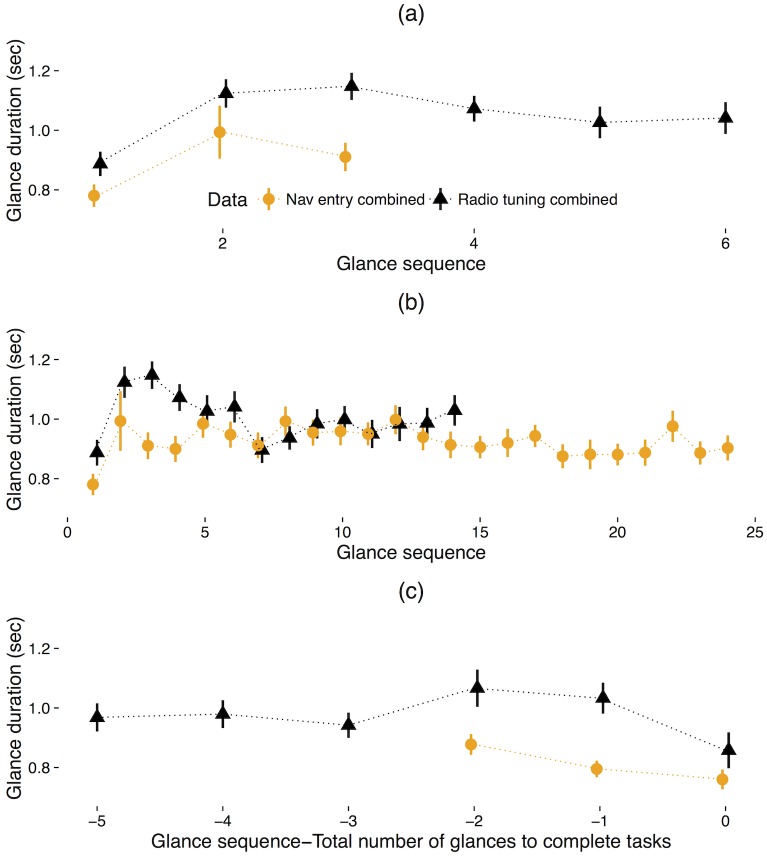
Glance duration comparison between the radio tuning tasks and navigation entry tasks showing (a) first six/three glances, (b) first 14/24 glances, and (c) last six/three glances.

Scenario 1 and 2 showed varying patterns of glance duration with respect to glance sequence; effect sizes as represented by marginal *R*^2^ were modest ranging from .008 to .02. However, given that different sequence lengths were employed in the analysis of each task, direct comparison of their linear models was not reasonable (Fig 11). As such, to compare the two tasks, the first three glances and the last three glances from the radio tuning tasks were reanalyzed and compared to the original analysis of the first three glances and the last three glances from the navigation entry tasks. Note that this is a less comprehensive assessment of sequence effects for the radio tuning task.

Results showed that there was a significant effect of glance sequence on duration, *F* (1, 77.63) = 26.05, *p* < .001, marginal *R*^*2*^ = .05, conditional *R*^*2*^ = .32, in the first three glances of the radio tuning tasks, indicating that a one unit increase in sequence is associated a 0.13 second increase in the glance duration. In addition, there was a significant effect of glance sequence in the last three glances of the radio tuning tasks, *F* (1, 77.77) = 9.25, *p* < .01, marginal *R*^*2*^ = .02, conditional *R*^*2*^ = .32, indicating that a one unit increase in glance sequence is associated a 0.10 second decrease in the glance duration. These patterns are similar to findings from the navigation entry tasks in Scenario 2.

The KS tests showed that the first glances’ duration was significantly different than other glances for both radio tuning tasks and navigation entry tasks (Tables 1 and 5). However, the last glances’ duration was significantly different for the radio tuning tasks, whereas in the navigation entry tasks, no statistical difference was observed.

## Discussion

This study analyzed the effect of glance sequence on glance behavior from 80 participants with double-coded, mediated, manually annotated region of interest glance data. The study applied multiple statistical techniques across the entire data set to compare not only mean differences, but also distributions of each glance sequence across a range of analytical approaches.

For the radio tuning tasks, according to mixed effects modeling, there were no significant patterns in the first six glances—the minimum number of glances to complete the task—and the last six glances. For the first 14 glances—the average number of glances to complete the task, the mixed effects modeling indicated that glance duration decreased as glance sequence increased. In addition, the KS tests found that the first and last glances were shorter than other glances.

For the navigation entry tasks, according to mixed effects modeling, significant patterns were observed from the last three glances—the minimum number of glances to complete the task—as glance duration decreased as glance sequence increased. However, the same was not the case for the first three and first 24 glances—the average number of glances to complete the task, where the mixed effects modeling showed no significant effects. As for the KS tests, the first glance was found to be shorter than other glances. There were no significant results of the KS test for the last three glances. In general, the discrepancy in results across the mixed effects modeling and KS tests may be attributed to the fact that the mixed effects modeling focuses on *average* values whereas the KS tests analysis considers the *distribution* of values.

By comparing results from mixed effects models varying the number of glances (first six vs. last six, and first three vs. last three) for the radio tuning task, we found that the results change depending on the number of glances. For example, no significant effect was observed when the first six and last six glances were tested, whereas significant effects were found when the first three and last three glances were tested for the same task type. This might be due to small effect sizes that we found from the first three (*R*^*2*^ = .05) and last three glances (*R*^*2*^ = .02) from the radio tuning task.

Lastly, the pattern of shorter first glances was not consistent across the two data sets for the radio tuning tasks; data set A showed shorter first glances where data set B did not. In terms of the radio tuning tasks, as this study utilized two data sets that differed in only the first button interaction, a deeper investigation as to how one action (e.g., pressing a power button) affects glance sequence and glance duration was considered. In essence, the seemingly slight difference in task characteristics significantly influenced glance duration. For example, with data set A, the duration of the first glance was shorter than the other five, whereas data set B showed no differences across glance sequence. Although there was a difference in the number of button presses between data sets A and B, a second important difference between the data sets is the size of the first button pressed (a relatively large, circular on/off/volume button vs. a smaller rectangular button in a row of similar buttons). Even though it is hard to provide concrete evidence as to whether the first glance was for finding the target button or for executing the first action (e.g., pressing the button), it is clear that glance duration might be dependent on characteristics of the task. Also, given that consistent patterns in the first glance were observed over the 80 participant group (e.g., data set A and B together) across the two different tasks by the KS test, it could be assumed that in general “first” glances may have a greater tendency to include some task independent characteristic—possibly an involvement in localization—that affects glance duration. Further investigation with higher resolution eye tracking may assist in untangling this hypothesis.

### Comparison with past research

In this study, data were drawn from drivers’ performance during a well-established visual-manual radio tuning task [15,19] and an auditory-vocal based multi-modal navigation entry task, both of which have significant ecological validity. Unlike past studies where more experimentally crafted tasks were completed in a driving simulator [10,11], this effort looked at glance sequence behaviors under on-road driving conditions using production systems. The tasks were generally longer in duration and performed in a self-paced and non-time limited format. While it is unknown how the pressure of a time limit influences glance behavior, it is plausible that results reported in [11] could be influenced by this artificial experimental characteristic. It is quite plausible to expect that the distribution of glance allocations would vary across tasks. To develop a deeper understanding of the phenomena, this study analyzed different types of DVI tasks separately to develop an understanding of how the construction of a task may influence glance sequence. This is similar to past studies [10], while different from others that included different types of tasks and collapsed them together for a single analysis [11]. While there are methodological differences between this research and past studies [10,11], a consistent theme across all of the work suggests that under various conditions, differences in duration across glance sequences appear. This research extends beyond earlier work by illustrating that other characteristics of an interface, such as control size, location, or salience, may be contributing factors influencing glance behavior.

### Limitations and future work

Though this work sheds light into the study of the effect of glance sequence on glance duration, a few limitations warrant future work. This study focused on glance behavior associated with two types of tasks. Future research should investigate the effect of glance behavior on other tasks of various duration and complexity as well as across greater variations in physical and structural interface characteristics. Research may need to consider a more fine-grained analysis of the sub-tasks associated with various activities and the relationship of DVI attributes (e.g., button size, haptic interfaces, and text size). For example, analysis of the individual sub-task could identify exactly what participants were doing during each glance. Lastly, the effect of glance sequence on glance behavior could be modulated by other factors. Demographic considerations, individual differences, susceptibility to distraction, roadway environment, and even the level of vehicle automation need to be assessed in future work to further uncover what prevailing factors influence the prediction of glance duration over the time course of DVI interactions.

## Conclusion

The present study applied multiple statistical approaches to test the effect of glance sequence on glance duration using double coded, mediated, manually coded region of interest based glance data from on-road driving. The only consistent finding across task types and statistical methods was that the first glance (for both task types) was significantly shorter than other glances. However, in the radio tuning task, this significant finding was largely driven by data set A, which required participants to push a large, salient button first. After analyzing the two data sets independently, results showed that data set A exhibited an effect of glance sequence on glance duration, whereas data set B did not. As the key difference between these two data sets was the added step of pressing a power button in data set A, the findings provide support for the effect of an interface’s physical characteristics on glance duration. As such, this study cannot confirm the consistent and replicable existence of a sequential effect on glance duration, but highlights the importance of considering the impact of interface and task characteristics on driver attention.

## Supporting information

S1 Data (ZIP)
